# *rac*-*N*-{6-[Bromo­(hydr­oxy)meth­yl]-2-pyrid­yl}pivalamide

**DOI:** 10.1107/S1600536809006795

**Published:** 2009-02-28

**Authors:** James C. Knight, Fawaz A. Saad, Angelo J. Amoroso, Benson M. Kariuki, Simon J. Coles

**Affiliations:** a Main Building, School of Chemistry, Cardiff University, Park Place, Cardiff CF10 3AT, Wales; b School of Chemistry, University of Southampton, Highfield, Southampton SO17 1BJ, England

## Abstract

The title compound, C_11_H_15_BrN_2_O_2_, contains an amide group which is close to coplanar with the adjacent pyridine ring, the dihedral angle between the planes being 9.0 (5)°. The mol­ecular packing reveals a mutual hydrogen-bond inter­action between centrosymmetrically related hydroxyl O atoms. Further hydrogen bonding involving O—H⋯Br and N—H⋯Br inter­actions also appears to consolidate the packing.

## Related literature

For a related structure, see: Goswami *et al.* (2005 ▶). For the synthesis, see Harata *et al.* (1995 ▶).
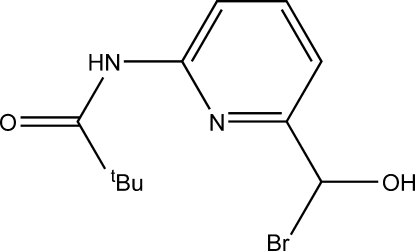


## Experimental

### 

#### Crystal data

C_11_H_15_BrN_2_O_2_
*M* *_r_* = 287.16
Monoclinic, 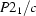

*a* = 13.2980 (5) Å
*b* = 10.0848 (3) Å
*c* = 9.4890 (3) Å
β = 106.858 (1)°
*V* = 1217.86 (7) Å^3^
*Z* = 4
Mo *K*α radiation
μ = 3.36 mm^−1^
*T* = 150 K
0.10 × 0.08 × 0.08 mm

#### Data collection

Bruker–Nonius KappaCCD diffractometer
Absorption correction: multi-scan (*SORTAV*; Blessing, 1995 ▶) *T*_min_ = 0.730, *T*_max_ = 0.771
11933 measured reflections
2773 independent reflections
2142 reflections with *I* > 2σ(*I*)
*R*_int_ = 0.081

#### Refinement

*R*[*F* ^2^ > 2σ(*F* ^2^)] = 0.060
*wR*(*F* ^2^) = 0.166
*S* = 1.10
2773 reflections
149 parameters
H-atom parameters constrained
Δρ_max_ = 0.53 e Å^−3^
Δρ_min_ = −0.99 e Å^−3^

### 

Data collection: *COLLECT* (Nonius, 2000 ▶); cell refinement: *SCALEPACK* (Otwinowski & Minor, 1997 ▶); data reduction: *DENZO* (Otwinowski & Minor, 1997 ▶) and *SCALEPACK*; program(s) used to solve structure: *SIR92* (Altomare *et al.*, 1993 ▶); program(s) used to refine structure: *SHELXL97* (Sheldrick, 2008 ▶); molecular graphics: *X-SEED* (Barbour, 2001 ▶); software used to prepare material for publication: *WinGX* (Farrugia, 1999 ▶) and *CHEMDRAW Ultra* (Cambridge Soft 2001).

## Supplementary Material

Crystal structure: contains datablocks I, global. DOI: 10.1107/S1600536809006795/sj2584sup1.cif

Structure factors: contains datablocks I. DOI: 10.1107/S1600536809006795/sj2584Isup2.hkl

Additional supplementary materials:  crystallographic information; 3D view; checkCIF report

## Figures and Tables

**Table 1:** Hydrogen-bond geometry (Å, °)

*D*—H⋯*A*	*D*—H	H⋯*A*	*D*⋯*A*	*D*—H⋯*A*
O1—H1*A*⋯O1^i^	0.84	2.03	2.472 (10)	113
O1—H1*A*⋯Br1^ii^	0.84	2.85	3.509 (5)	137
N2—H2⋯Br1^ii^	0.88	2.97	3.690 (4)	140

Symmetry codes: (i) 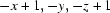; (ii) 
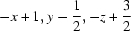
.

**Fig. 1.**
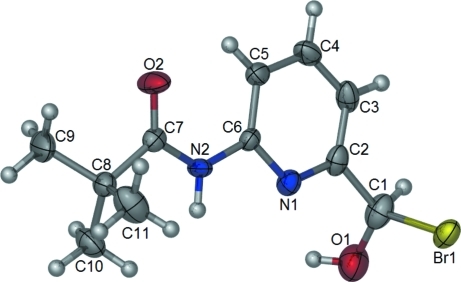
Molecular structure of the title compound, showing the atom numbering. Displacement ellipsoids are drawn at the 50% probablility level. H atoms are represented by circles of arbitrary size.

**Fig. 2.**
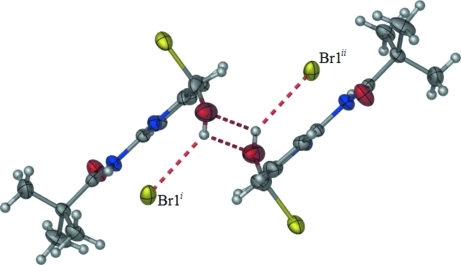
A view showing the mutual H-bond interaction between centrosymmetrically related hydroxyl oxygen atoms O1 and O1i. A short H···H distance is suggestive of unresolved synchronized disorder between these groups. The hydrogen atom is also involved in H-bonding with a bromine acceptor atom located on a different molecule. [symmetry code (i): 1 - x, y - 1/2, 3/2 - z.]

**Fig. 3.**
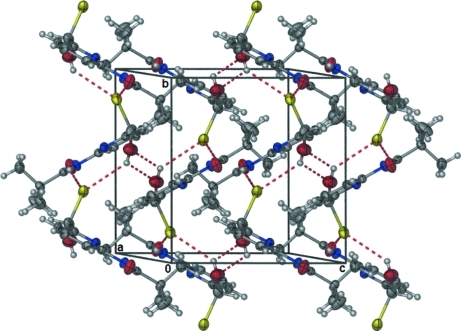
A view of the molecular packing. H-bonding interactions are indicated by a dashed line. Displacement ellipsoids are drawn at the 50% probablility level. H atoms are represented by circles of arbitrary size.

**Fig. 4.**
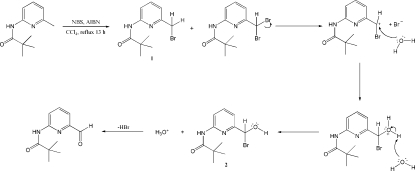
Preparation of the title compound.

## supplementary crystallographic information

### Comment

During research focused on new synthetic routes towards novel poly-pyridyl co-ordination compounds, we observed an unexpected by-product in one of our syntheses. As we attempted to prepare *N*-(6-(bromomethyl)pyridin-2-yl)pivalamide (1) from *N*-(6-methylpyridin-2-yl)pivalamide and *N*-bromosuccinimide (NBS) in the presence of azobisisobutyronitrile (AIBN) (Harata *et al.*, 1995), the title compound (2) was isolated in low yield (Fig. 3).

We postulate that during the free-radical driven mono-bromination reaction (Scheme 2), a small quantity of *N*-(6-(dibromomethyl)pyridin-2-yl)pivalamide is generated. The formation of 2 would proceed *via* an S_N_1 reaction involving water, arising as a minor contaminant within the solvent. Subsequent elimination of HBr would lead to the formation of the corresponding aldehyde, however, we have isolated this intermediate prior to HBr elimination.

The formation of the stereogenic centre (C1) from achiral starting materials without any optically active agent has naturally led to a racemic compound. The geometry surrounding the C1 atom is a distorted tetrahedron, which supports the *sp*^3^-hybridization of this carbon, and the comparatively long C1–O1 bond (1.440 (8) Å) confirms the presence of an alcohol rather than a ketone. Additionally, while no hydroxyl proton was observed in the ^1^H NMR, the spectrum revealed a diagnostic singlet at a considerable downfield shift of 6.40 p.p.m. which, according to the ^1^H NMR prediction software (*CHEMDRAW* Ultra 8.0; Cambridge Soft 2001), is indicative of a proton (H1) in an α-position to a hydroxyl group and a bromine atom. The single-crystal infrared spectrum of this compound also features just a single band in the carbonyl region (1694.0 cm^-1^) attributable to the amide carbonyl stretch. The bond lengths and angles of the title compound are in good agreement with the expected values (Goswami *et al.*, 2005).

The mutual H-bond interaction between hydroxyl oxygen atoms O1 and O1^i^ (Fig. 2) results in a short H···H distance (1.87 Å) which is indicative of some disorder between the hydroxyl groups. Attempts at modeling this disorder have given unsatisfactory results. See Table 1 for details of other H-bond interactions which support the crystal packing (Fig. 3).

### Experimental

*N*-(6-methylpyridin-2-yl)pivalamide (40 g, 0.207 mol), NBS (56 g, 0.315 mol), and a catalytic amount of AIBN were dissolved in carbon tetrachloride (400 ml). The reaction mixture was heated at reflux for 13 h. The resulting crude brown oil was purified *via* column chromatography (hexane:ethyl acetate (95:5)). Recrystallization from hexane afforded crystals (yield 1.7%) suitable for X-ray diffraction. ^1^H NMR (400 MHz; CDCl_3_): 7.94 (*s*, 1H, H2), 7.92 (*d*, 1H, *J* = 7.87 Hz, py-H5), 7.70 (*t*, 1H, *J* = 7.89 Hz, py-H4), 7.37 (*d*, 1H, *J* = 7.60 Hz, py-H3), 6.40 (*s*, 1H, H1), 1.27 (*s*, 9H, H9–11). IR (KBr disk): 1694.0 cm^-1^ (—C=O). HRMS (EI) *m/z*: calc. for C_11_H_15_BrN_2_O 270.0368, found 270.0373.

### Refinement

H atoms attached to C, N and O atoms were placed in calculated positions and subsequently treated as riding with C—H distances of 0.95–1.00 Å, an N—H distance of 0.88 Å, and an O—H distance of 0.84 Å. The *U*_iso_(H) was set to be 1.5*U*eq of the carrier atom for hydroxyl and methyl H atoms, and *U*_iso_(H) = 1.2*U*eq(C) for all other H atoms. The deepest hole in electron density (-0.99 e A^-3^) is located at the distance of 0.36 Å from O1.

### Crystal data

**Table:**

C_11_H_15_BrN_2_O_2_	*F*(000) = 584
*M**_r_* = 287.16	*D*_x_ = 1.566 Mg m^−^^3^
Monoclinic, *P*2_1_/*c*	Mo *K*α radiation, λ = 0.71073 Å
Hall symbol: -P 2ybc	Cell parameters from 9199 reflections
*a* = 13.2980 (5) Å	θ = 2.9–27.5°
*b* = 10.0848 (3) Å	µ = 3.36 mm^−^^1^
*c* = 9.4890 (3) Å	*T* = 150 K
β = 106.858 (1)°	Prism, colourless
*V* = 1217.86 (7) Å^3^	0.10 × 0.08 × 0.08 mm
*Z* = 4	

### Data collection

**Table:**

Bruker–Nonius KappaCCD diffractometer	2773 independent reflections
Radiation source: fine-focus sealed tube	2142 reflections with *I* > 2σ(*I*)
graphite	*R*_int_ = 0.081
Detector resolution: 9 pixels mm^-1^	θ_max_ = 27.5°, θ_min_ = 3.0°
φ and ω scans	*h* = −17→17
Absorption correction: multi-scan (*SORTAV*; Blessing, 1995)	*k* = −13→13
*T*_min_ = 0.730, *T*_max_ = 0.771	*l* = −12→12
11933 measured reflections	

### Refinement

**Table:**

Refinement on *F*^2^	Secondary atom site location: difference Fourier map
Least-squares matrix: full	Hydrogen site location: inferred from neighbouring sites
*R*[*F*^2^ > 2σ(*F*^2^)] = 0.060	H-atom parameters constrained
*wR*(*F*^2^) = 0.166	*w* = 1/[σ^2^(*F*_o_^2^) + (0.0702*P*)^2^ + 2.6431*P*] where *P* = (*F*_o_^2^ + 2*F*_c_^2^)/3
*S* = 1.10	(Δ/σ)_max_ = 0.001
2773 reflections	Δρ_max_ = 0.53 e Å^−^^3^
149 parameters	Δρ_min_ = −0.99 e Å^−^^3^
0 restraints	Extinction correction: *SHELXL97* (Sheldrick, 2008), Fc^*^=kFc[1+0.001xFc^2^λ^3^/sin(2θ)]^-1/4^
Primary atom site location: structure-invariant direct methods	Extinction coefficient: 0.011 (2)

### Special details

**Table:**

Geometry. All e.s.d.'s (except the e.s.d. in the dihedral angle between two l.s. planes) are estimated using the full covariance matrix. The cell e.s.d.'s are taken into account individually in the estimation of e.s.d.'s in distances, angles and torsion angles; correlations between e.s.d.'s in cell parameters are only used when they are defined by crystal symmetry. An approximate (isotropic) treatment of cell e.s.d.'s is used for estimating e.s.d.'s involving l.s. planes.
Refinement. Refinement of *F*^2^ against ALL reflections. The weighted *R*-factor *wR* and goodness of fit *S* are based on *F*^2^, conventional *R*-factors *R* are based on *F*, with *F* set to zero for negative *F*^2^. The threshold expression of *F*^2^ > σ(*F*^2^) is used only for calculating *R*-factors(gt) *etc*. and is not relevant to the choice of reflections for refinement. *R*-factors based on *F*^2^ are statistically about twice as large as those based on *F*, and *R*- factors based on ALL data will be even larger.

### Fractional atomic coordinates and isotropic or equivalent isotropic displacement parameters (Å^2^)

**Table:**

	*x*	*y*	*z*	*U*_iso_*/*U*_eq_	
C1	0.4573 (5)	0.1876 (6)	0.5557 (7)	0.0503 (14)	
H1	0.4497	0.2093	0.4503	0.060*	
C2	0.3456 (4)	0.1520 (5)	0.5509 (5)	0.0377 (11)	
C3	0.2616 (5)	0.1806 (5)	0.4307 (5)	0.0420 (12)	
H3	0.2718	0.2221	0.3460	0.050*	
C4	0.1622 (4)	0.1473 (5)	0.4365 (6)	0.0428 (12)	
H4	0.1027	0.1675	0.3558	0.051*	
C5	0.1492 (4)	0.0846 (5)	0.5600 (5)	0.0357 (11)	
H5	0.0814	0.0615	0.5662	0.043*	
C6	0.2389 (3)	0.0566 (4)	0.6742 (5)	0.0285 (9)	
C7	0.1548 (3)	−0.0513 (4)	0.8487 (5)	0.0304 (10)	
C8	0.1806 (4)	−0.1281 (5)	0.9947 (5)	0.0344 (11)	
C9	0.0890 (5)	−0.2212 (7)	0.9885 (8)	0.0646 (19)	
H9A	0.1061	−0.2764	1.0772	0.097*	
H9B	0.0763	−0.2780	0.9013	0.097*	
H9C	0.0259	−0.1689	0.9829	0.097*	
C10	0.2806 (5)	−0.2096 (6)	1.0243 (6)	0.0511 (14)	
H10A	0.3407	−0.1503	1.0345	0.077*	
H10B	0.2750	−0.2707	0.9421	0.077*	
H10C	0.2906	−0.2603	1.1154	0.077*	
C11	0.1918 (6)	−0.0255 (7)	1.1159 (6)	0.0621 (17)	
H11A	0.2044	−0.0707	1.2109	0.093*	
H11B	0.1271	0.0268	1.0961	0.093*	
H11C	0.2511	0.0332	1.1189	0.093*	
N1	0.3360 (3)	0.0902 (4)	0.6727 (4)	0.0344 (9)	
N2	0.2386 (3)	−0.0119 (4)	0.8029 (4)	0.0319 (9)	
H2	0.3009	−0.0323	0.8622	0.038*	
O1	0.5396 (4)	0.0896 (5)	0.5873 (5)	0.0644 (12)	
H1A	0.5149	0.0157	0.6012	0.097*	
O2	0.0655 (3)	−0.0259 (4)	0.7793 (4)	0.0514 (10)	
Br1	0.49376 (4)	0.36048 (6)	0.65166 (6)	0.0461 (3)	

### Atomic displacement parameters (Å^2^)

**Table:**

	*U*^11^	*U*^22^	*U*^33^	*U*^12^	*U*^13^	*U*^23^
C1	0.054 (3)	0.056 (3)	0.051 (3)	−0.015 (3)	0.031 (3)	−0.010 (3)
C2	0.048 (3)	0.033 (3)	0.036 (2)	−0.012 (2)	0.019 (2)	−0.007 (2)
C3	0.061 (3)	0.036 (3)	0.030 (2)	−0.011 (2)	0.015 (2)	0.001 (2)
C4	0.047 (3)	0.043 (3)	0.030 (2)	0.000 (2)	−0.001 (2)	0.005 (2)
C5	0.033 (2)	0.032 (2)	0.037 (3)	−0.003 (2)	0.003 (2)	0.000 (2)
C6	0.026 (2)	0.028 (2)	0.031 (2)	−0.0052 (17)	0.0074 (17)	−0.0011 (18)
C7	0.030 (2)	0.028 (2)	0.034 (2)	0.0002 (18)	0.0111 (19)	−0.0038 (19)
C8	0.030 (2)	0.038 (3)	0.036 (2)	−0.001 (2)	0.012 (2)	0.007 (2)
C9	0.045 (3)	0.068 (4)	0.081 (5)	−0.010 (3)	0.018 (3)	0.036 (4)
C10	0.050 (3)	0.054 (4)	0.049 (3)	0.009 (3)	0.015 (3)	0.019 (3)
C11	0.088 (5)	0.060 (4)	0.040 (3)	0.012 (4)	0.022 (3)	0.003 (3)
N1	0.033 (2)	0.039 (2)	0.034 (2)	−0.0047 (17)	0.0135 (17)	0.0003 (18)
N2	0.0209 (17)	0.043 (2)	0.0293 (19)	0.0010 (16)	0.0028 (14)	0.0090 (17)
O1	0.061 (3)	0.055 (3)	0.084 (3)	−0.004 (2)	0.032 (2)	0.004 (2)
O2	0.0238 (17)	0.083 (3)	0.046 (2)	−0.0002 (18)	0.0081 (15)	0.018 (2)
Br1	0.0413 (4)	0.0561 (4)	0.0409 (3)	−0.0175 (2)	0.0118 (2)	0.0002 (2)

### Geometric parameters (Å, °)

**Table:**

C1—O1	1.440 (8)	C7—C8	1.536 (7)
C1—C2	1.516 (7)	C8—C10	1.518 (7)
C1—Br1	1.962 (6)	C8—C11	1.522 (8)
C1—H1	1.0000	C8—C9	1.526 (7)
C2—N1	1.352 (6)	C9—H9A	0.9800
C2—C3	1.376 (8)	C9—H9B	0.9800
C3—C4	1.380 (8)	C9—H9C	0.9800
C3—H3	0.9500	C10—H10A	0.9800
C4—C5	1.386 (7)	C10—H10B	0.9800
C4—H4	0.9500	C10—H10C	0.9800
C5—C6	1.388 (6)	C11—H11A	0.9800
C5—H5	0.9500	C11—H11B	0.9800
C6—N1	1.339 (6)	C11—H11C	0.9800
C6—N2	1.404 (6)	N2—H2	0.8800
C7—O2	1.207 (6)	O1—H1A	0.8400
C7—N2	1.367 (6)		
			
O1—C1—C2	121.5 (5)	C10—C8—C7	113.2 (4)
O1—C1—Br1	116.2 (4)	C11—C8—C7	106.7 (4)
C2—C1—Br1	109.4 (4)	C9—C8—C7	107.9 (4)
O1—C1—H1	102.0	C8—C9—H9A	109.5
C2—C1—H1	102.0	C8—C9—H9B	109.5
Br1—C1—H1	102.0	H9A—C9—H9B	109.5
N1—C2—C3	123.4 (5)	C8—C9—H9C	109.5
N1—C2—C1	114.7 (5)	H9A—C9—H9C	109.5
C3—C2—C1	121.9 (5)	H9B—C9—H9C	109.5
C2—C3—C4	118.2 (5)	C8—C10—H10A	109.5
C2—C3—H3	120.9	C8—C10—H10B	109.5
C4—C3—H3	120.9	H10A—C10—H10B	109.5
C3—C4—C5	120.0 (5)	C8—C10—H10C	109.5
C3—C4—H4	120.0	H10A—C10—H10C	109.5
C5—C4—H4	120.0	H10B—C10—H10C	109.5
C4—C5—C6	117.6 (5)	C8—C11—H11A	109.5
C4—C5—H5	121.2	C8—C11—H11B	109.5
C6—C5—H5	121.2	H11A—C11—H11B	109.5
N1—C6—C5	123.6 (4)	C8—C11—H11C	109.5
N1—C6—N2	112.3 (4)	H11A—C11—H11C	109.5
C5—C6—N2	124.1 (4)	H11B—C11—H11C	109.5
O2—C7—N2	121.9 (4)	C6—N1—C2	117.2 (4)
O2—C7—C8	121.8 (4)	C7—N2—C6	128.9 (4)
N2—C7—C8	116.3 (4)	C7—N2—H2	115.6
C10—C8—C11	109.9 (5)	C6—N2—H2	115.6
C10—C8—C9	108.7 (5)	C1—O1—H1A	109.5
C11—C8—C9	110.4 (5)		
			
O1—C1—C2—N1	53.7 (7)	O2—C7—C8—C11	−88.7 (6)
Br1—C1—C2—N1	−86.3 (5)	N2—C7—C8—C11	90.4 (5)
O1—C1—C2—C3	−126.0 (6)	O2—C7—C8—C9	30.0 (7)
Br1—C1—C2—C3	93.9 (5)	N2—C7—C8—C9	−150.9 (5)
N1—C2—C3—C4	1.7 (8)	C5—C6—N1—C2	−1.6 (7)
C1—C2—C3—C4	−178.7 (5)	N2—C6—N1—C2	177.4 (4)
C2—C3—C4—C5	−1.2 (8)	C3—C2—N1—C6	−0.3 (7)
C3—C4—C5—C6	−0.5 (8)	C1—C2—N1—C6	−180.0 (4)
C4—C5—C6—N1	2.0 (7)	O2—C7—N2—C6	−2.2 (8)
C4—C5—C6—N2	−176.9 (5)	C8—C7—N2—C6	178.7 (4)
O2—C7—C8—C10	150.3 (5)	N1—C6—N2—C7	173.7 (4)
N2—C7—C8—C10	−30.6 (6)	C5—C6—N2—C7	−7.3 (8)

### Hydrogen-bond geometry (Å, °)

**Table:**

*D*—H···*A*	*D*—H	H···*A*	*D*···*A*	*D*—H···*A*
O1—H1A···O1^i^	0.84	2.03	2.472 (10)	113
O1—H1A···Br1^ii^	0.84	2.85	3.509 (5)	137
N2—H2···Br1^ii^	0.88	2.97	3.690 (4)	140

Symmetry codes: (i) −*x*+1, −*y*, −*z*+1; (ii) −*x*+1, *y*−1/2, −*z*+3/2.
